# Risk Factors for Unplanned Higher-Level Re-Amputation and Mortality after Lower Extremity Amputation in Chronic Limb-Threatening Ischemia

**DOI:** 10.3390/jcm13144020

**Published:** 2024-07-10

**Authors:** Andres Guerra, Michelle Guo, Riley M. Boyd, Marina Zakharevich, Andrew W. Hoel, Ashley K. Vavra, Jeanette W. Chung, Karen J. Ho

**Affiliations:** 1 Department of Surgery, Feinberg School of Medicine, Northwestern University, Chicago, IL 60611, USA; andres.guerra@nm.org (A.G.); michelle.guo1@northwestern.edu (M.G.); andrew.hoel@nm.org (A.W.H.); ashley.vavra@nm.org (A.K.V.); 2 Department of Pediatrics, Ann & Robert H. Lurie Children’s Hospital of Chicago, Chicago, IL 60611, USA; rmboyd@luriechildrens.org; 3 Department of Emergency Medicine, Stanford Medicine, Palo Alto, CA 94305, USA; mzakh@stanford.edu; 4 Department of Surgery, Indiana University School of Medicine, Indianapolis, IN 46202, USA; jeawchun@iu.edu

**Keywords:** retrospective study, lower extremity, logistic models, odds ratio, confidence intervals, risk factors, lower extremity, amputation surgical, re-amputation, peripheral artery disease, chronic limb-threatening ischemia

## Abstract

**Background:** The factors associated with unplanned higher-level re-amputation (UHRA) and one-year mortality among patients with chronic limb-threatening ischemia (CLTI) after lower extremity amputation are poorly understood. **Methods:** This was a single-center retrospective study of patients who underwent amputations for CLTI between 2014 and 2017. Unadjusted bivariate analyses and adjusted odds ratios (AOR) from logistic regression models were used to assess associations between pre-amputation risk factors and outcomes (UHRA and one-year mortality). **Results:** We obtained data on 203 amputations from 182 patients (median age 65 years [interquartile range (IQR) 57, 75]; 70.7% males), including 118 (58.1%) toe, 20 (9.9%) transmetatarsal (TMA), 37 (18.2%) below-knee (BKA), and 28 (13.8%) amputations at or above the knee. Median follow-up was 285 days (IQR 62, 1348). Thirty-six limbs (17.7%) had a UHRA, and the majority of these (72.2%) were following index forefoot amputations. Risk factors for UHRA included non-ambulatory status (AOR 6.74, 95% confidence interval (CI) 1.74–26.18; *p* < 0.10) and toe pressure < 30 mm Hg (AOR 4.89, 95% CI 1.52–15.78; *p* < 0.01). One-year mortality was 17.2% (n = 32), and risk factors included coronary artery disease (AOR 3.93, 95% CI 1.56–9.87; *p* < 0.05), congestive heart failure (AOR 4.90, 95% CI 1.96–12.29; *p* = 0.001), end-stage renal disease (AOR 7.54, 95% CI 3.10–18.34; *p* < 0.001), and non-independent ambulation (AOR 4.31, 95% CI 1.20–15.49; *p* = 0.03). Male sex was associated with a reduced odds of death at 1 year (AOR 0.37, 95% CI 0.15–0.89; *p* < 0.05). UHRA was not associated with one-year mortality. **Conclusions:** Rates of UHRA after toe amputations and TMA are high despite revascularization and one-year mortality is high among patients with CLTI requiring amputation.

## 1. Introduction

Chronic limb-threatening ischemia (CLTI) is a severe form of lower extremity peripheral artery disease (PAD) that is associated with a 15–20% rate of one-year lower extremity amputation and 15–40% rate of one-year mortality [1,2]. Amputees experience poor quality of life secondary to decreased mobility, social isolation, and chronic pain [3]. This impact is exacerbated by secondary procedures, particularly unplanned higher-level re-amputation (UHRA). The reported risk of re-amputation varies between 10.7% and 43.2% and depends on level of index amputation and a variety of patient- and limb-specific characteristics [4,5,6,7,8,9,10,11]. Retrospective cohort studies using clinical registries and administrative databases lack granular patient-specific characteristics (such as preoperative ambulatory status) and clinical characteristics (such as wound status or hemodynamic measures) that factor into clinical decision-making at the time of amputation.

The aims of this study were to characterize the risk of UHRA in patients with CLTI using a single-center dataset with detailed patient- and limb-level data. Secondary outcomes included mortality and a composite outcome of return to the operating room or UHRA. We sought to identify risk factors with the goal of improving surgical decision-making and prognostic accuracy.

## 2. Materials and Methods

### 2.1. Study Population

We conducted a single-center retrospective cohort study of patients who underwent primary lower extremity amputation for CLTI in the Division of Vascular Surgery at Northwestern Memorial Hospital in Chicago, IL between 1 January 2014 and 31 December 2017. Cases were excluded if amputations were performed for other indications (including acute limb ischemia or trauma) or if the primary amputation was an open ankle disarticulation performed for control of sepsis with planned future revision to a higher-level amputation. All open toe amputations performed for infection were included unless a higher-level amputation was planned a priori. A waiver of written informed consent was obtained, and patient information was abstracted from the electronic medical record after approval from the Northwestern University Institutional Review Board (STU00210604).

All patients underwent a thorough clinical examination by one of seven board-certified attending vascular surgeons for lower extremity symptoms, ischemia, infection, and wounds. Each surgeon individually determined the appropriate type of index amputation based on severity of symptoms, physical exam findings, extent of tissue loss (if present), non-invasive studies (ankle brachial index [ABI], segmental pressures with waveforms, toe pressure), and imaging studies (computed tomography angiography [CTA], magnetic resonance angiography [MRA], or invasive diagnostic angiography). Index amputations were either at the toe, transmetatarsal (TMA), transtibial or below-knee (BKA), through-knee (TKA), or transfemoral or above-knee (AKA) level. Each surgeon also individually determined the indications for UHRA based on wound and patient characteristics, including failure of healing of the index amputation.

### 2.2. Definition of Terms

Tissue loss is an ulceration that does not heal for at least three months. Excisional debridement is surgical removal of non-healing tissue in the operating room. An UHRA is an amputation performed after the index amputation at a more proximal level that includes a joint. For toe amputations, a higher-level re-amputation is a transmetatarsal or more proximal amputation. Infection was defined as the presence of purulence, cellulitis, wet gangrene, or exposed bone with systemic signs including constitutional symptoms and leukocytosis or imaging findings consistent with deep space infection or osteomyelitis. ABI was considered invalid due to non-compressible vessels if ≥1.4.

### 2.3. Study Outcomes

The primary outcome was UHRA after index amputation. Secondary outcomes were death within one year of index amputation and a composite endpoint of return to the operating room for wound closure, revision, excisional debridement, revascularization, or UHRA.

### 2.4. Data Collection

Variables were categorized as patient-level or limb-level. Patient-level variables included demographics (age, sex, race, and ethnicity), medical history, and medications. Limb-level variables included wound, ischemia, and foot infection classification (WIfI) [12], hemodynamic data (ABI, toe pressures, and ankle Doppler waveforms), and angiographic data. Risk of limb amputation was assigned as very low to low (stage 1–2) or moderate to high (stage 3–4) according to WIfI classification [12]. Ankle waveforms were classified as “multiphasic” if both dorsalis pedis and posterior tibial artery ankle waveforms were multiphasic or “abnormal” if either artery at the ankle had a monophasic or absent waveform. CTA, MRA, and/or diagnostic angiography was used to evaluate the patency of lower extremity arteries and the presence or absence of genicular arteries (Supplemental Figure S1).

### 2.5. Statistical Analysis

Cohort characteristics were summarized as percentages for categorical data and mean ± standard deviation (SD) or median and interquartile range (IQR) for continuous data. Unadjusted bivariate associations between each risk factor and each outcome were examined using Fisher’s exact tests for categorical variables or Student’s *t*-tests for continuous variables. Adjusted associations between clinical risk factors and each outcome were estimated using logistic regression models. All models were adjusted for index amputation type, patient age, race (white [reference] vs. non-white) and sex (male vs. female [reference]). Some patients had multiple amputations in the same limb during the follow-up period. These re-amputations were “pooled” in order to increase the observation sample size. A separate analysis included bivariate and multivariable regression performed with the pooled dataset on UHRA and the composite endpoint with adjunctive tables is presented in Supplemental Table S1. Regression analyses were performed with Huber–White standard errors to account for clustering at the patient level. Due to the modest sample size and potential collinearity between some clinical risk factors, separate models were estimated for each clinical risk factor and outcome.

Adjusted associations between clinical risk factors and time to death were estimated using Cox proportional hazards models. Time to death was measured in days from index amputation to death, and the censoring variable was death within one year. Models adjusted for index amputation type, patient age, race (white [reference] vs. non-white) and sex (male vs. female [reference]). Variables with insufficient variation were excluded from the regression models. These included history of hypertension and the patency of the common femoral artery, profunda femoris artery, and descending branch of the lateral circumflex artery. Missing data were handled using case-wise deletion (complete cases analyzed). The estimation sample sizes (number of limbs) for each risk factor model are shown in Supplemental Table S2. The proportional hazards assumption was evaluated for each model. We present *p* values unadjusted for multiple comparisons and denote associations that remain significant (*p* < 0.0012) after Bonferroni correction for multiple testing with over 40 comparisons. Stata^®^ Statistical Software: Release 18 (StataCorp LLC, College Station, TX) was used for statistical analysis.

## 3. Results

### 3.1. Characteristics of the Study Population

A total of 182 patients underwent 203 lower extremity primary amputations during the study period. The median length of follow-up was 285 days (IQR 62, 1378). Patient-level characteristics and types of index amputation are presented in Table 1A and Table 1B, respectively. The median age was 65 years (IQR 57, 75) and most patients were male (70.3%) and had diabetes mellitus (70.8%). Only 56.0% of patients were on optimal medical treatment for PAD consisting of antiplatelet and lipid-lowering therapy [13] at the time of index amputation.

Of the 188 limbs with waveform studies prior to index amputation, most had multiphasic ipsilateral femoral and popliteal artery waveforms (97.9% [n = 184] and 88.8% [n = 167], respectively). One-hundred and one limbs (53.7%) had multiphasic waveforms in both ipsilateral ankle arteries and 46.3% (n = 87) had at least one “abnormal” ankle waveform. Seventy-seven limbs (40.5%) had invalid ABI due to calcified vessels; of those with valid ABI (n = 113), 49.6% (n = 56) had diminished ABI (median ABI 0.8 [0.5, 1.0]). Of the limbs that were assessed with toe pressures (80.3%, n = 163), 55.2% (n = 90) were ≥30 mm Hg, 11.7% (n = 19) were < 30 mm Hg, and 33.1% (n = 54) were absent. For the majority of patients who underwent revascularization either concomitant with or after index amputation, non-invasive vascular testing was performed prior to index amputation and does not reflect the revascularized state.

Of the limbs that had pre-operative ipsilateral cross-sectional imaging (73.9%, n = 150), 96.0% (n = 144) had a patent common femoral artery and 95.3% (n = 143) had a patent profunda femoris artery. Of the limbs with angiographic imaging that included the ipsilateral knee and calf (67.5%, n = 137), 59.1% (n = 81) had either four or five of the six genicular arteries present (Supplemental Figure S1).

### 3.2. UHRA

Thirty-six limbs (17.7%) went on to undergo a subsequent UHRA. Median time to UHRA was 47 days (IQR 25.5, 87.5). Most UHRA occurred after toe amputation (n = 26, 22.0%) or TMA (n = 6, 30.0%) (*p* = 0.02). All unadjusted bivariate analyses are shown in Table 2. Although male patients were more susceptible to UHRA compared to female patients (21.4% vs. 8.8%, *p* = 0.04), there was not a statistically significant difference on multivariable analysis (Table 3). Limbs with ipsilateral revascularization prior to, or concurrent with, index amputation were at higher risk of UHRA compared to limbs without revascularization in the bivariate analysis (25.3% vs. 12.5%, respectively; *p* = 0.03). Technical success was achieved in 100% of the procedures. Of the 87 limbs that had ipsilateral revascularization, 35 (40.2%) and 52 (59.8%) underwent open and endovascular revascularization, respectively. Of the open revascularization procedures, 8 (22.9%) were associated with subsequent UHRA while 52 (26.9%) of the endovascular procedures were associated with subsequent UHRA (*p* = 0.70). Similarly, limbs that underwent revascularization after index amputation were at higher risk of UHRA compared to limbs that were not revascularized after index amputation (34.5% vs. 14.9%, respectively; *p* = 0.02). However, revascularization was not significantly associated with UHRA on multivariable analysis (Table 3). There was also no difference in UHRA based on indication for index amputation, WIfI stage of amputation risk, presence of diabetes mellitus or end-stage renal disease (ESRD), or the number of patent genicular vessels present on pre-operative angiography with UHRA. Patients with an ipsilateral abnormal ankle waveform had 3.12 greater odds (95% confidence interval [CI] 1.30–7.46) of undergoing UHRA (Table 3). Furthermore, patients with ipsilateral toe pressures ≤ 30 mm Hg at the time of index amputation had greater odds of UHRA compared to those with either toe pressures ≥ 30 mm Hg or absent toe pressures (OR 4.89; 95% confidence interval [95% CI] 1.52–15.78, *p* < 0.01), suggesting that more ischemic limbs were more likely to have a definitive primary amputation. In addition, patients who underwent excisional debridement after index amputation had 4.18 greater odds (95% CI 1.13–15.50, *p* < 0.05) of UHRA. 

### 3.3. Secondary Outcomes

Of the 182 patients, 32 (17.6%; corresponding to 36 limbs) died within one year of index amputation. On bivariate analysis of mortality, females were more predisposed to death within one year compared to male patients (28.1% vs. 13.8%, respectively; *p* = 0.02). Comorbidities associated with increased risk of death at one year included coronary artery disease (CAD) (27.2% vs. 10.6% without, *p* = 0.01), history of myocardial infarction (30.3% vs. 15.34% without, *p* = 0.05), congestive heart failure (CHF) (35.9% vs. 11.1% without, *p* < 0.001), and ESRD (34.9% vs. 9.7% without, *p* < 0.001). Patients with moderate/high risk of amputation within one year based on WIfI were also at higher risk of death within one year (20.9% vs. 5.6% with very/low risk, *p* = 0.05). A relatively low fraction of patients were on optimal medical therapy at the time of index amputation but this was not associated with either the primary or secondary outcomes.

On multivariable analysis of mortality, male patients had a lower hazard of death at one year compared to female patients (HR 0.41, 95% CI 0.20–0.83) (Table 4). Several patient factors were associated with greater hazard of death, including need for assistance with ambulation (HR 3.76, 95% CI 1.11–12.78), CHF (HR 3.51, 95% CI 1.64–7.51), and ESRD (HR 5.35, 95% CI 2.51–11.43). There was no significant association between UHRA and death at one year (23.5% UHRA vs. 16.6% without UHRA; *p* = 0.27).

Risk factors associated with return to the operating room identified by bivariate analysis included male sex (46.2% of males vs. 28.1% of females; *p* = 0.03). Limbs with ABI between 0.5 and 0.8 had a higher risk for reoperation, while limbs with an ABI ≤ 0.5 had the lowest risk for reoperation (64.3% vs. 21.4%, respectively; *p* = 0.01), suggesting that the index amputation was more likely to be definitive in severely ischemic limbs. Not surprisingly, limbs with partially open or open index amputations had 2.5 greater odds (95% CI 1.18–5.23) of the composite secondary outcome on multivariable analysis (Table 3). There was no statistically significant association between patient level factors or pre-operative limb level factors with the composite secondary outcome (Table 3).

### 3.4. Pooled Data Outcomes

Pooling all amputations, including multiple amputations on the same limb, we observed 300 amputations during study period. In analyses of pooled limb-level data, 26 patients (14.3%) underwent bilateral amputations during the study period. Of these, 27 limbs (13.3%) underwent a second re-amputation, and 8 limbs (3.9%) underwent a third re-amputation. In the pooled dataset, risk factors for UHRA included statin, antiplatelet, or anticoagulant use, non-ambulatory status, toe pressure < 30 mm Hg, and amputations left partially closed or open (Supplemental Table S1). Patients with a toe pressure < 30 mm Hg had higher odds of returning to the operating room (OR 2.97, 95% CI 1.22–7.24) compared to patients with toe pressure ≥ 30 mmHg.

## 4. Discussion

In this single-center cohort study of patients with CLTI undergoing lower extremity amputation, the rate of re-amputation was 17.7% and mortality one year after index amputation was 17.2%. Risk factors for mortality included presence of CAD, CHF, ESRD, female sex, need for assistance with ambulation at the time of index amputation.

The rates of UHRA we observed concur with published rates of re-amputation in patients with PAD. Czerniecki et al. [10] reported an overall 24.4% rate of re-amputation among patients with diabetes and/or PAD in the Veterans Health Administration. Lin et al. [11] reported 17% and 23% rates of re-amputation after minor amputation for patients with PAD and both PAD and diabetes mellitus, respectively. Among patients in the VA Surgical Quality Improvement Program database with PAD and/or diabetes, the rates of re-amputation were 9–40% depending on the level of the index amputation.

Variation in rate of re-amputation based on level of index amputation, including a higher rate of re-amputation associated with a more distal index operation, is also consistent with the literature. Our observed UHRA rates of 30%, 8.1%, and 3.6% after TMA, BKA and TKA, or AKA, respectively, were less than the respective re-amputation rates of approximately 40%, 25%, and 9%, reported by Czerniecki et al. [10] and Norvell & Czerniecki [9] but followed a similar pattern. While we observed a rate of 22.0% for UHRA after toe amputation, Collins et al. [8] reported a rate of 43%, and Yammine et al. [14] reported a rate of 51.2% for re-amputation after toe amputation.

Reported time to UHRA is variable, but we found relatively shorter time to UHRA compared to others, which may be due to differences in definition of re-amputation endpoints. Whereas we found a median time to UHRA of 47 days (IQR 2.5–87.5), Norvell & Czerniecki [9] reported a median of 33 (IQR 13–73) days, Birmpili et al. [4] reported a median of 76 (IQR 20–344) days (minor amputation to major re-amputation), Lin et al. [11] reported a median of 4.9 months (IQR = 1.8–14.7) (second minor re-amputation) and 12.9 months (IQR 5.4–27.6) (minor amputation to major re-amputation), and Collins [8] reported a median of 36 months (minor amputation to second minor or major re-amputation). These data highlight the prolonged time course associated with recovery from amputations of any level, and the time from index amputation to UHRA may be characterized by repeated clinic and wound care visits for poor healing, pain, limited mobility, dependent care, and limited potential for ambulation [9].

In conducting this single-center retrospective study, we were well-positioned to examine both traditional risk factors for re-amputation (such as demographic factors and medical history) and the associations of other patient- and limb-level details, such as ambulatory status, living situation, presence of patent collateral arteries, and hemodynamic measures, with UHRA. After multivariable adjustment, we found UHRA to be positively associated with index amputation level, non-ambulatory status, toe pressure < 30 mm Hg and abnormal ankle waveform at the time of index amputation, and debridement after index amputation. We found that revascularization was significantly associated with increased UHRA on bivariate analysis, suggesting surgeon bias at the time of revascularization towards a lower threshold for considering these limbs for salvage. However, revascularization was not a significant factor in the logistic regression model, suggesting a need for investigation in a larger dataset. This contrasts with the findings of Lin et al. [11], who found that those who underwent revascularization before a repeat minor amputation had a significantly decreased risk of a major amputation compared to those who were intervened on after a repeat minor amputation.

One-year mortality after lower extremity amputation in patients with PAD has been estimated as approximately 20% [4,8], similar to our finding of 17.2%. Like others, we found that the presence of comorbidities such as CAD, CHF, and ESRD were associated with one-year mortality [4]. Novel findings in our study that were associated with mortality included other patient-level factors such as ambulatory status at index operation and limb-level factors such as presence of infection and invalid ABI. The latter finding is consistent with the observation that among all adults with PAD, the presence of non-compressible vessels is associated with increased mortality compared to vessels with a valid ABI [15]. Since most re-amputations occurred after index forefoot amputations, we cannot determine if survival after UHRA is associated with the level of re-amputation.

This study had several limitations. First, it was a single-center cohort with a modest sample size; hence, the logistic regression model included a limited number of variables. For example, we could not control for variability in the decision-making of individual surgeons in our practice. Second, some variables (e.g., patent collateral arteries and non-invasive measures of blood flow) were not available for all patients. Other variables that were not available for enough of the cohort were not included. For example, we were unable to assess whether perioperative hemoglobin A1c, an index of glycemic control, was associated with UHRA. Third, some variables had insufficient variation in our sample. This might not be a problem in a larger and/or multi-site dataset. Fourth, these data came from a single academic medical center and involved patients from seven surgeons. Consequently, the generalizability of our findings may be limited. As a tertiary academic center, our sample may have included a higher proportion of patients with advanced ischemic disease, complex wounds, a history of failed revascularization and/or patients seeking additional opinions for limb salvage compared to non-academic medical centers. The relatively low fraction of patients on optimal medical therapy at the time of index amputation is of historical significance; contemporary Vascular Quality Initiative-based data from our center demonstrates > 90% compliance at the time of discharge with optimal medical therapy. Finally, we did not have amputation status at the time of death and hence cannot assess if patients who died within one year were more likely to refuse UHRA, which would have biased the results.

## 5. Conclusions

In summary, we found that rates of UHRA after toe amputations and TMA are high despite revascularization. Patients with CLTI requiring amputation, regardless of subsequent UHRA, are at high risk of one-year mortality. Future research using larger and/or multi-center datasets and hierarchical modeling of surgeon-, patient-, and limb-level factors may reveal further insights that could be used to optimize pre-amputation risk factors and identify amputees at high risk of UHRA.

## Figures and Tables

**Table 1:** A. Patient-Level Characteristics at Time of Index Amputation. B. Index Amputations by Type.

	N (%)
(A)	
Median age, years (IQR)	65 (57, 75)
Sex (n = 182)	
Female	54 (29.3)
Male	128 (70.7)
Race (n = 173)	
White	89 (51.5)
Non-white	84 (48.6)
Ambulatory status (n = 182)	
Ambulatory	60 (33.0)
Ambulatory with assistance	91 (50.0)
Non-ambulatory	31 (17.0)
Living status (n = 178)	
Home	162 (91.0)
Other	16 (9.0)
Medical history	
Stroke (n = 182)	18 (9.9)
Coronary artery disease (n = 177)	81 (45.8)
Myocardial infarction (n = 177)	29 (16.4)
Congestive heart failure (n = 178)	46 (25.8)
Hypertension (n = 178)	160 (89.9)
Hyperlipidemia (n = 178)	86 (48.3)
Atrial fibrillation (n = 178)	38 (21.4)
Chronic obstructive pulmonary disease (n = 178)	24 (13.5)
End-stage renal disease (n = 178)	55 (30.9)
Diabetes mellitus (n = 178)	126 (70.8)
Malignancy (n = 178)	26 (14.6)
Smoking (n = 180)	
Never	74 (41.1)
Former	79 (43.9)
Current	27 (15.0)
Medications	
Statin (n = 178)	130 (73.0)
Antiplatelet (n = 178)	122 (68.5)
Anticoagulation (n = 178)	53 (29.8)
Dual anti-platelet (n = 178)	37 (20.8)
Optimal medical therapy (n = 182)	102 (56.0)
(B)	
Toe	118 (58.1)
Transmetatarsal	20 (9.9)
Below-knee	37 (18.2)
Through- or above-knee	28 (13.8)

n in left column represents number of participants with valid observations. Data in right column are presented as frequencies with percentages in parentheses.

**Table 2:** Patient and Limb Characteristics by UHRA.

Patient and Limb Characteristic	No UHRA 167 Limbs (82.3%)	UHRA 36 Limbs (17.7%)	*p* Value
Age, mean (SD) ^[a]^	65.40 (12.5)	64.72 (13.0)	0.79
Sex			0.04
Female	52 (91.2)	5 (8.8)	
Male	114 (78.6)	31 (21.4)	
Race			0.58
Non-white	82 (83.7)	16 (16.3)	
White	75 (80.0)	19 (20.2)	
Ambulatory status			0.65
Ambulatory	57 (86.4)	9 (13.6)	
Ambulatory with assistance	81 (81.0)	19 (19.0)	
Non-ambulatory	29 (80.6)	7 (19.4)	
Living situation			0.70
Home	147 (82.6)	31 (17.4)	
Other	15 (80.0)	4 (21.1)	
Medical history			
Stroke	17 (85.0)	3 (15.0)	1.00
Coronary artery disease	70 (77.8)	20 (22.2)	0.17
Myocardial infarction	28 (84.9)	5 (15.2)	0.81
Congestive heart failure	41 (77.4)	12 (22.6)	0.30
Hypertension	144 (81.8)	32 (18.2)	1.00
Hyperlipidemia	74 (79.6)	19 (20.4)	0.46
Atrial fibrillation	32 (74.4)	11 (25.6)	0.17
Chronic obstructive pulmonary disease	22 (75.9)	7 (24.1)	0.36
End-stage renal disease	52 (82.5)	11 (17.5)	1.00
Diabetes mellitus	116 (81.7)	26 (18.3)	0.84
Malignancy	23 (82.1)	5 (17.9)	1.00
Smoking status			0.44
Never	65 (81.3)	15 (18.8)	
Former	71 (79.8)	18 (20.2)	
Current	29 (90.6)	3 (9.4)	
Medications			
Statin	114 (79.7)	29 (20.3)	0.15
Antiplatelet	107 (80.5)	26 (19.6)	0.37
Anticoagulation	45 (73.8)	16 (26.2)	0.05
Dual antiplatelet	29 (76.3)	9 (23.7)	0.34
Optimal medical therapy	90 (79.7)	23 (20.4)	0.30
Non-invasive vascular testing			
Waveform—femoral			1.00
Multiphasic	149 (81.0)	35 (19.0)	
Abnormal	4 (100.0)	0 (0.0)	
Waveform—popliteal			0.60
Multiphasic	135 (80.8)	32 (19.2)	
Abnormal	18 (85.7)	3 (14.3)	
Waveform—ankle			0.12
Multiphasic	87 (86.1)	14 (13.9)	
Abnormal	67 (77.0)	20 (23.0)	
ABI			0.24
0.8–1.4	47 (82.5)	10 (17.5)	
0.5–0.8	19 (67.9)	9 (32.1)	
≤0.5	25 (89.3)	3 (10.7)	
Invalid	65 (84.4)	12 (15.6)	
Toe pressure			0.04
≥30 mm Hg	73 (81.1)	17 (18.9)	
<30 mm Hg	11 (57.9)	8 (42.1)	
0 mm Hg	47 (87.0)	7 (13.0)	
Angiographic findings			
Patent common femoral artery	115 (79.9)	29 (20.1)	0.99
Patent profunda femoris artery	115 (80.4)	28 (19.6)	0.26
Patent genicular arteries, n (%)			0.77
4 or 5	64 (79.0)	17 (21.0)	
3 or fewer	43 (76.8)	13 (23.2)	
WIfI 1-year amputation risk			0.99
Very low or low risk	29 (80.6)	7 (19.4)	
Moderate or high risk	104 (80.6)	25 (19.4)	
Index amputation type			0.03
Toe	92 (78.0)	26 (22.0)	
Transmetatarsal	14 (70.0)	6 (30.0)	
Below-knee	34 (92.0)	3 (8.1)	
Through- or above-knee	27 (96.4)	1 (3.6)	
Indication for index amputation			0.27
Dry gangrene	62 (75.6)	20 (24.4)	
Any infection	77 (87.5)	11 (12.5)	
Nonhealing wound or rest pain	28 (84.9)	5 (15.2)	
Infection present	117 (85.4)	20 (14.6)	0.12
Index amputation, open or closed			0.70
Closed	85 (83.3)	17 (16.7)	
Partially open or open	82 (81.2)	19 (18.8)	
Ipsilateral revascularization prior to or concomitant with index amputation	65 (74.7)	22 (25.3)	0.03
Revascularization after index amputation	19 (65.5)	10 (34.5)	0.01
Debridement after index amputation	14 (66.7)	7 (33.3)	0.05

Data presented as frequencies with percentages in parentheses. ABI, ankle-brachial index; WIfI, wound, infection, and ischemia index. *p* values unadjusted for multiple comparisons. Fisher’s exact test except where noted (^[a]^ Student’s *t*-test). All tests two-tailed. All variables measured at index.

**Table 3:** Logistic Regression Estimates of the Association between Risk Factors and Outcomes Following Index Amputation.

	Adjusted Odds Ratios (95% CI)		
	UHRA (n = 36)	Death within One Year (n = 36)	Composite (n = 74)
Age	1.01 (0.98–1.04)	1.01 (0.98–1.04)	0.98 (0.95–1.00) †
Male (Ref: Female)	2.58 (0.93–7.17) †	0.37 (0.15–0.89) *	1.48 (0.73–3.01)
Non-white (Ref: White)	1.48 (0.70–3.11)	0.63 (0.28–1.44)	0.99 (0.52–1.86)
Ambulatory status (Ref: Ambulatory)			
Ambulatory with assistance	2.36 (0.86–6.51) †	4.31 (1.20–15.49) *	1.52 (0.72–3.21)
Non-ambulatory	6.74 (1.74–26.18) **	4.13 (0.80–21.39) †	1.44 (0.54–3.87)
Living status (Ref: Home)			
Other	0.97 (0.28–3.44)	0.40 (0.67–2.41)	0.80 (0.26–2.49)
Medical history			
Stroke (Ref: No)	0.84 (0.20–3.42)	0.43 (0.08–2.30)	1.24 (0.46–3.36)
Coronary artery disease (Ref: No)	2.00 (0.88–4.58)	3.93 (1.56–9.87) **	0.91 (0.48–1.72)
Myocardial infarction (Ref: No)	0.91 (0.34–2.39)	2.22 (0.86–5.76)	1.50 (0.67–3.39)
Congestive heart failure (Ref: No)	1.36 (0.61–3.04)	4.90 (1.96–12.29) **	0.88 (0.43–1.84)
Hyperlipidemia (Ref: No)	1.42 (0.64–3.15)	0.91 (0.41–2.00)	1.01 (0.53–1.93)
Atrial fibrillation (Ref: No)	1.56 (0.68–3.59)	2.49 (0.82–7.54)	0.55 (0.25–1.21)
Chronic obstructive pulmonary disease (Ref: No)	2.31 (0.87–6.08) †	2.26 (0.73–6.98)	1.17 (0.44–3.12)
End-stage renal disease (Ref: No)	1.07 (0.46–2.48)	7.54 (3.10–18.34) *** ^‡^	0.97 (0.46–2.04)
Diabetes mellitus (Ref: No)	0.78 (0.32–1.94)	1.05 (0.41–2.69)	0.77 (0.34–1.73)
Malignancy (Ref: No)	0.93 (0.35–2.51)	1.61 (0.61–4.30)	1.61 (0.64–4.07)
Smoking Status (Ref: Never)			
Former	1.10 (0.46–2.65)	0.53 (0.22–1.28)	1.22 (0.59–2.52)
Current	0.37 (0.09–1.55)	1.05 (0.26–4.15)	1.18 (0.39–3.53)
Medications			
Statin (Ref: No)	2.39 (0.84–6.78)	1.73 (0.65–4.61)	1.57 (0.76–3.24)
Antiplatelet (Ref: No)	2.02 (0.78–5.24)	1.03 (0.45–2.37)	0.74 (0.37–1.47)
Anticoagulation (Ref: No)	3.33 (1.43–7.72) **	0.70 (0.26–1.93)	1.43 (0.69–2.98)
Dual antiplatelet (Ref: No)	1.87 (0.71–4.93)	1.74 (0.72–4.17)	0.94 (0.45–2.00)
Optimal medical therapy (Ref: No)	1.92 (0.83–4.48)	1.34 (0.59–3.06)	0.88 (0.46–1.68)
Non-invasive vascular testing			
Abnormal waveform—femoral (Ref: Multiphasic)	-----	1.15 (0.08–17.35)	-----
Abnormal waveform—popliteal (Ref: Multiphasic)	1.53 (0.44–7.14)	1.79 (0.48–6.73)	1.07 (0.40–2.88)
Abnormal waveform—ankle (Ref: Multiphasic)	3.12 (1.30–7.46) *	1.59 (0.67–3.77)	1.53 (0.75–3.13)
ABI (Ref: 0.8–1.4)			
0.5–0.8	2.88 (0.79–10.48)	1.82 (0.45–7.39)	1.16 (0.41–3.32)
≤0.5	2.76 (0.50–15.16)	0.87 (0.17–4.44)	0.61 (0.19–1.99)
Invalid	0.97 (0.33–2.80)	4.79 (1.44–15.29) *	0.71 (0.30–1.65)
Toe pressure (Ref: ≥30 mm Hg)			
<30 mm Hg	4.89 (1.52–15.78) **	2.82 (0.75–10.66)	1.18 (0.37–3.75)
0 mm Hg	1.16 (0.36–3.73)	3.34 (0.98–11.36) †	0.74 (0.29–1.86)
Angiographic findings Patent genicular arteries (Ref: 4 or 5)			
≤3	1.11 (0.45–2.75)	0.47 (0.16–1.35)	1.43 (0.65–3.19)
WIfI 1-year amputation risk (Ref: Very low/low risk)			
Moderate/high risk	1.13 (0.37–3.46)	4.55 (0.94–22.10) †	1.89 (0.80–4.51)
Index amputation type (Ref: Toe)			
TMA	1.64 (0.51–5.23)	2.39 (0.64–8.88)	0.66 (0.19–2.23)
BKA	0.22 (0.05–0.98) *	1.00 (0.36–2.81)	0.34 (0.15–0.74) **
TKA or AKA	0.15 (0.02–1.15) †	1.28 (0.42–3.93)	0.22 (0.09–0.58) **
Indication (Ref: Dry gangrene)			
Any infection	0.42 (0.17–1.03) †	0.35 (0.13–0.90) *	1.08 (0.52–2.27)
Nonhealing wound or rest pain	1.18 (0.32–4.33)	0.73 (0.21–2.62)	0.82 (0.33–2.09)
Infection (Ref: None)	0.35 (0.15–0.78) *	1.31 (0.48–3.52)	0.72 (0.35–1.50)
Partially open or open index amputation (Ref: Closed)	0.57 (0.25–1.29)	1.00 (0.38–2.65)	2.49 (1.18–5.23) *
Revascularization prior to or concomitant to index amputation (Ref: No)	2.19 (0.88–5.49) †	0.81 (0.35–1.86)	0.89 (0.46–1.71)
Revascularization after index amputation (Ref: No)	2.16 (0.89–5.24) †	0.45 (0.12–1.65)	-----
Debridement after index amputation (Ref: No)	4.18 (1.13–15.50) *	0.47 (0.10–2.26)	-----
UHRA (Ref: No)	-----	2.00 (0.76–5.25)	-----

N = 203 index amputations. N in estimation sample may be smaller due to case-wise deletion of missing data and/or small cells. CI, confidence interval; TMA, transmetatarsal amputation; BKA, below-knee amputation; TKA, through-knee amputation; AKA, above-knee amputation; ABI, ankle-brachial index; WIfI, wound, infection, and ischemia index. †, *p* < 0.10; *, *p* < 0.05; **, *p* < 0.01; ***, *p* < 0.001. All variables measured at index amputation. Parameter estimates are from logistic regression models controlling for amputation type, sex, race, and age. All other patient characteristics were included in models for each one separately due to the small sample size. ^‡^, remains significant after Bonferroni correction for 37 tests (implies lowering *p* < 0.05 to *p* < 0.0014).

**Table 4:** Cox Regression Estimates of the Association between Risk Factors and Hazard of Death within One Year.

Patient Characteristic	Hazard Ratio (95% CI)	*p* Value
Age	1.00 (0.98–1.03)	0.75
Male (Ref: Female)	0.41 (0.20–0.83)	0.01
Non-white (Ref: White)	0.68 (0.33–1.37)	0.28
Ambulatory status at index (Ref: Ambulatory)		
Ambulatory with assistance	3.76 (1.11–12.78)	0.03
Non-ambulatory	3.65 (0.78–17.03)	0.10
Living situation (Ref: Home)		
Other	0.44 (0.08–2.37)	0.34
Medical history		
Stroke (Ref: No)	0.45 (0.10–1.98)	0.29
Coronary artery disease (Ref: No)	2.97 (1.31–6.71)	0.01
Myocardial infarction (Ref: No)	1.91 (0.86–4.26)	0.11
Congestive heart failure (Ref: No)	3.51 (1.64–7.51)	0.001
Hyperlipidemia (Ref: No)	0.90 (0.45–1.80)	0.76
Atrial fibrillation (Ref: No)	2.32 (0.78–6.86)	0.13
Chronic obstructive pulmonary disease (Ref: No)	1.98 (0.78–5.05)	0.15
End-stage renal disease (Ref: No)	5.35 (2.51–11.43) ^‡^	<0.001
Malignancy (Ref: No)	1.46 (0.62–3.46)	0.39
Smoking status (Ref: Never)		
Former	0.61 (0.27–1.35)	0.22
Current	1.09 (0.31–3.78)	0.89
Medications		
Statin (Ref: No)	1.59 (0.66–3.87)	0.30
Antiplatelet (Ref: No)	1.00 (0.47–2.13)	1.00
Anticoagulation (Ref: No)	0.73 (0.30–1.78)	0.49
Dual antiplatelet (Ref: No)	1.85 (0.85–4.03)	0.12
Optimal medical therapy (Ref: No)	1.32 (0.63–2.73)	0.46
Non-invasive vascular testing		
Abnormal waveform—femoral (Ref: Multiphasic)	0.93 (0.11–7.77)	0.95
Abnormal waveform—popliteal (Ref: Multiphasic)	1.57 (0.49–5.04)	0.45
Abnormal waveform—ankle (Ref: Multiphasic)	1.35 (0.62–2.94)	0.45
ABI (Ref: 0.8–1.4)		
0.5–0.8	1.55 (0.44–5.42)	0.50
≤0.5	0.82 (0.19–3.60)	0.79
Invalid	3.48 (1.17–10.36)	0.03
Toe pressure (Ref: ≥30 mm Hg)		
<30 mm Hg	2.56 (0.78–8.42)	0.12
0 mm Hg	2.61 (0.89–7.68)	0.08
Angiographic findings Patent genicular arteries (Ref: 4 or 5)		
≤3	0.54 (0.21–1.39)	0.20
WIfI 1-year amputation risk (Ref: Very/low risk)		
Moderate/high risk	3.88 (0.84–17.80)	0.08
Amputation type (Ref: Toe)		
Transmetatarsal	1.86 (0.68–5.05)	0.23
Below-knee	0.97 (0.38–2.46)	0.95
Through- or above-knee	1.26 (0.49–3.24)	0.63
Indication (Ref: Dry gangrene)		
Any infection	0.40 (0.17–0.96)	0.04
Nonhealing wound or rest pain	0.78 (0.24–2.57)	0.68
Infection (Ref: None)	1.29 (0.52–3.22)	0.58
Partially open or open (Ref: Closed)	0.99 (0.40–2.45)	0.99
Revascularization prior to or concomitant to index amputation (Ref: No)	0.81 (0.36–1.80)	0.60
Revascularization after index amputation (Ref: No)	0.51 (0.16–1.65)	0.26
Debridement after index amputation (Ref: No)	0.51 (0.12–2.12)	0.35
UHRA	1.87 (0.80–4.37)	0.24

N = 203 index amputations (ankle disarticulation excluded). N in estimation sample may be smaller due to case-wise deletion of missing data and/or small cells. CI, confidence interval; ABI, ankle-brachial index; WIfI, wound, infection, and ischemia index. All variables measured at index. Parameter estimates are from Cox regression models controlling for amputation type, sex, race, and age. All other patient characteristics were included in models for each one separately due to the small sample size. ^‡^, remains significant after Bonferroni correction for 35 tests (implies lowering *p* < 0.05 to *p* < 0.0014).

## Data Availability

These data are available only upon request due to patient confidentiality concerns. All requests need to be vetted to determine whether benefits outweigh risks and to assess appropriateness in accordance with institutional data sharing restrictions.

## Supplementary Materials

The following supporting information can be downloaded at: https://www.mdpi.com/article/10.3390/jcm13144020/s1, Figure S1: Representative lower extremity digital subtraction angiography; Table S1: Logistic Regression Estimates of the Association between Risk Factors and Patient Outcomes Following Index Amputation—Pooled Dataset; Table S2: Estimation Sample Sizes for Logistic Regression Estimates of the Association between Risk Factors and Outcomes Following Index Amputation as Reported in Table 3.
