# Supplementary material for: Risk Factors for Unplanned Higher-Level Re-Amputation and Mortality after Lower Extremity Amputation in Chronic Limb-Threatening Ischemia
Source: J Clin Med. 2024 Jul 10;13(14):4020. doi: 10.3390/jcm13144020 (PMC11277533; doi:10.3390/jcm13144020)
Supplement: Supplementary file 1 [file jcm-13-04020-s001.zip › jcm-3015286-supplementary.pdf]

SUPPLEMENTARY MATERIALS

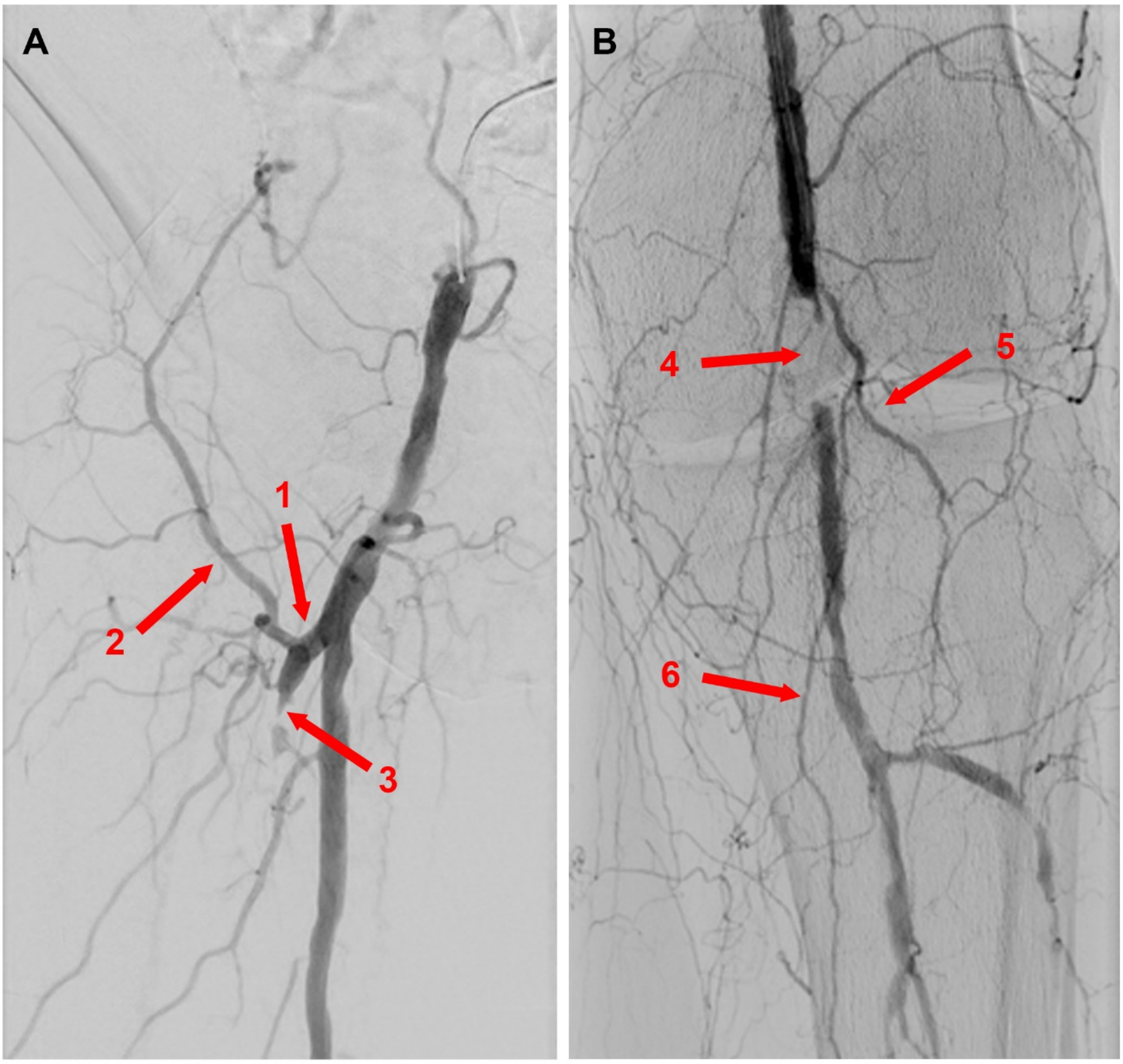

Figure S1. Representative lower extremity digital subtraction angiography. A. Angiogram of a patient with a patent profunda femoris artery (1), patent lateral femoral circumflex artery (2), and occluded descending branch of the lateral femoral circumflex artery (3). B. Angiogram of a patient with an occluded behind-the-knee popliteal artery (4) with patent genicular vessels (5 and 6) reconstituting the below-knee popliteal segment.

Table S1. Logistic Regression Estimates of the Association between Risk Factors and Patient Outcomes Following Index Amputation - Pooled Dataset

	Adjusted Odds Ratios (95% CI)	
**Patient Characteristic**	**UHRA (n=58)**	**Composite (n=115)**
Age	1.00 (0.98-1.02)	0.99 (0.97-1.01)
Male (Ref: Female)	1.93 (0.87-4.31)	1.71 (0.95-3.04)†
Nonwhite (Ref: White)	1.34 (0.75-2.40)	1.17 (0.71-1.90)
Ambulatory status (Ref: Ambulatory)		
Ambulatory with assistance	2.21 (0.95-5.12)†	1.46 (0.80-2.64)
Non-ambulatory	3.48 (1.14-10.63)*	1.14 (0.50-2.56)
Living status: Other (Ref: Home)	0.75 (0.31-1.81)	0.71 (0.32-1.55)
Medical history		
Stroke (Ref: No)	1.10 (0.37-3.27)	1.35 (0.70-2.63)
Coronary artery disease (Ref: No)	1.63 (0.87-3.08)	1.29 (0.78-2.16)
Myocardial infarction (Ref: No)	0.65 (0.28-1.51)	0.80 (0.40-1.58)
Congestive heart failure (Ref: No)	1.25 (0.67-2.35)	0.99 (0.59-1.65)
Hyperlipidemia (Ref: No)	1.59 (0.87-2.91)	1.25 (0.77-2.03)
Atrial fibrillation (Ref: No)	1.51 (0.77-2.95)	0.73 (0.40-1.33)
COPD (Ref: No)	1.60 (0.79-3.21)	1.45 (0.78-2.68)
End-stage renal disease (Ref: No)	0.92 (0.47-1.80)	1.07 (0.62-1.86)
Diabetes mellitus (Ref: No)	1.02 (0.53-1.93)	0.91 (0.53-1.57)
Malignancy (Ref: No)	1.13 (0.56-2.28)	1.21 (0.59-2.48)
Smoking Status (Ref: Never)		
Former	1.23 (0.66-2.31)	1.27 (0.74-2.19)
Current	0.42 (0.16-1.10)†	0.87 (0.38-2.01)
Medications		
Statin (Ref: No)	3.29 (1.51-7.17)**	2.02 (1.14-3.57)*
Antiplatelet (Ref: No)	2.56 (1.18-5.56)*	1.45 (0.86-2.46)
Anticoagulation (Ref: No)	1.96 (1.05-3.68)*	1.27 (0.76-2.13)
Dual antiplatelet (Ref: No)	1.84 (0.91-3.73)†	0.77 (0.44-1.35)
Optimal medical therapy (Ref: No)	2.72 (1.34-5.54)**	1.51 (0.92-2.48)
Non-invasive vascular testing		
Abnormal waveform – femoral (Ref: Multiphasic)	-----	-----
Abnormal waveform – popliteal (Ref: Multiphasic)	0.77 (0.21-2.84)	1.74 (0.71-4.24)
Abnormal waveform – ankle (Ref: Multiphasic)	2.10 (1.11-3.97)*	2.00 (1.15-3.47)
ABI (Ref: 0.8-1.4)		
0.5-0.8	1.83 (0.72-4.67)	2.06 (0.88-4.80)†
≤0.5	2.94 (0.92-9.41)†	1.74 (0.68-4.41)
Non compressible	1.28 (0.60-2.73)	1.54 (0.84-2.81)
Toe pressure (Ref: ≥30 mm Hg)		
<30 mm Hg	5.39 (2.22-13.10)*** ‡	2.97 (1.22-7.24)*
0 mm Hg	0.95 (0.44-2.04)	1.02 (0.53-1.94)
Angiographic findings		
Patent genicular arteries: ≤3 (Ref: 4 or 5)	0.91 (0.47-1.76)	0.96 (0.54-1.70)

	Adjusted Odds Ratios (95% CI)	
**Patient Characteristic**	**UHRA (n=58)**	**Composite (n=115)**
WIFI 1-yr amputation risk (Ref: Very low or low risk)		
Moderate or high risk	1.16 (0.60-2.25)	1.12 (0.59-2.14)
Index amputation type (ref: Toe)		
Transmetatarsal	1.36 (0.62-2.99)	1.98 (0.90-4.38)†
Below-knee	0.26 (0.09-0.77)*	0.95 (0.49-1.81)
Through- or above-knee	0.18 (0.05-0.69)*	0.61 (0.29-1.28)
Indication (Ref: Dry gangrene)		
Any infection	0.49 (0.23-1.03)†	1.08 (0.61-1.93)
Nonhealing wound or rest pain	1.00 (0.38-2.64)	1.43 (0.69-2.98)
Closed index amputation (Ref: Partially open or open)	0.51 (0.27-0.93)*	1.06 (0.62-1.81)
Revascularization prior to or concomitant to index amputation (Ref: None)	1.52 (0.79 – 2.93)	1.11 (0.64 – 1.91)
Revascularization after index amputation (Ref: None)	2.16 (0.89 - 5.24)†	------
Debridement after index amputation (Ref: No)*	2.92 (1.21-7.04)*	-----
UHRA	-----	-----

Estimates are from logistic regression analyses with Huber-White standard errors. CI, confidence interval; ABI, ankle-brachial index; WIFI, wound, infection, and ischemia index; UHRA, unplanned higher-level re-amputation. P values unadjusted for multiple comparisons, †, p<0.10; *, p<0.05; **, p<0.01; ***, p<0.001. ‡, Result remains significant at p<0.05 after Bonferroni adjustment for 35 tests (which implies lowering p<0.05 to p<0.001)

Table S2: Estimation Sample Sizes for Logistic Regression Estimates of the Association between Risk Factors and Outcomes Following Index Amputation as Reported in Table 3

	UHRA	Death Within One Year	Composite
Age	n=192	n=192	n=192
Sex	n=192	n=192	n=192
Race	n=192	n=192	n=192
Ambulatory status	n=191	n=191	n=191
Living situation	n=192	n=192	n=192
Medical history			
Stroke	n=192	n=192	n=192
Coronary artery disease	n=192	n=192	n=192
Myocardial infarction	n=192	n=192	n=192
Congestive heart failure	n=192	n=192	n=192
Hyperlipidemia	n=192	n=192	n=192
Atrial fibrillation	n=192	n=192	n=192
Chronic obstructive pulmonary disease	n=192	n=192	n=192
End-stage renal disease	n=192	n=192	n=192
Diabetes mellitus	n=192	n=192	n=192
Malignancy	n=192	n=192	n=192
Smoking status	n=190	n=190	n=190
Medications			
Statin	n=192	n=192	n=192
Antiplatelet	n=192	n=192	n=192
Anticoagulation	n=192	n=192	n=192
Dual antiplatelet	n=192	n=192	n=192
Optimal medical therapy	n=192	n=192	n=192
Non-invasive vascular testing			
Abnormal waveform – femoral	n=154	n=177	n=173
Abnormal waveform – popliteal	n=156	n=177	n=177
Abnormal waveform – ankle	n=159	n=178	n=178
ABI	n=159	n=179	n=179
Toe pressure	n=140	n=155	n=155
Angiographic findings			
Patent genicular arteries	n=133	n=133	n=133
WIFI 1-yr amputation risk	n=141	n=157	n=157
Amputation type	n=192	n=192	n=192
Indication	n=192	n=185	n=192
Infection	n=192	n=192	n=192

	UHRA	Death Within One Year	Composite
Open/closed	n=192	n=192	n=192
Revascularization prior to or concomitant with index amputation	n=189	n=189	n=189
Revascularization after index amputation	n=192	n=192	n=163
Debridement	n=192	n=192	n=172
UHRA	-----	n=192	-----

ABI, ankle-brachial index; WIFI, wound, infection, and ischemia index; UHRA, unplanned higher-level re-amputation.
